# *Myristica fragrans* Kernels Prevent Paracetamol-Induced Hepatotoxicity by Inducing Anti-Apoptotic Genes and Nrf2/HO-1 Pathway

**DOI:** 10.3390/ijms20040993

**Published:** 2019-02-25

**Authors:** Mohamed A. Dkhil, Ahmed E. Abdel Moneim, Taghreed A. Hafez, Murad A. Mubaraki, Walid F. Mohamed, Felwa A. Thagfan, Saleh Al-Quraishy

**Affiliations:** 1 Department of Zoology, College of Science, King Saud University, Riyadh 11451, Saudi Arabia; guraishi@yahoo.com; 2 Department of Zoology and Entomology, Faculty of Science, Helwan University, Cairo 11795, Egypt; 3 Clinical Laboratory Sciences Department, College of Applied Medical Sciences, King Saud University, Riyadh 11433, Saudi Arabia; thafiz@KSU.EDU.SA (T.A.H.); mmubaraki@KSU.EDU.SA (M.A.M.); 4 Department of Biological and Geological Sciences, Faculty of Education, Ain Shams University, Cairo 11341, Egypt; walidfathy72@yahoo.com; 5 Department of Biology, College of Science, Princess Nourah bint Abdulrahman University, Riyadh 11671, Saudi Arabia; fafa-85@hotmail.com

**Keywords:** paracetamol, *Myristica fragrans* kernels, heme oxygenase 1, liver

## Abstract

Paracetamol is responsible for acute liver failure in humans and experimental animals when taken at high doses and transformed into a reactive metabolite by the liver cytochrome P450. On the other hand, nutmeg is rich with many phytochemical ingredients that are known for their ability to inhibit cytochrome P450. Hence, the present experiment was aimed at studying the hepatoprotective effect of *Myristica fragrans* (nutmeg), kernel extract (MFKE) in respect to paracetamol (acetaminophen; *N*-acetyl-p-amino-phenol (APAP))-induced hepatotoxicity in rats, focusing on its antioxidant, anti-inflammatory, and anti-apoptotic activities. Liver toxicity was induced in rats by a single oral administration of APAP (2 g/kg). To evaluate the hepatoprotective effect of MFKE against this APAP-induced hepatotoxicity, rats were pre-treated with either oral administration of MFKE at 300 mg/kg daily for seven days or silymarin at 50 mg/kg as a standard hepatoprotective agent. APAP intoxication caused a drastic elevation in liver function markers (transaminases, alkaline phosphatase, and total bilirubin), oxidative stress indicators (lipid peroxidation and nitric oxide), inflammatory biomarkers (tumour necrosis factor-α, interleukin-1β, inducible nitric oxide synthase, and nuclear factor ĸB) and the pro-apoptotic BCL2 Associated X (Bax) and caspases-3 genes. Furthermore, analyses of rat liver tissue revealed that APAP significantly depleted glutathione and inhibited the activities of antioxidant enzymes in addition to downregulating two key anti-apoptotic genes: Cellular FLICE (FADD-like IL-1β-converting enzyme)-inhibitory protein (c-FLIP) and B-cell lymphoma 2 (Bcl-2). Pre-treatment with MFKE, however, attenuated APAP-induced liver toxicity by reversing all of these toxicity biomarkers. This hepatoprotective effect of MFKE was further confirmed by improvement in histopathological findings. Interestingly, the hepatoprotective effect of MFKE was comparable to that offered by the reference hepatoprotector, silymarin. In conclusion, our results revealed that MFKE had antioxidant, anti-inflammatory, and anti-apoptotic properties, and it is suggested that this hepatoprotective effect could be linked to its ability to promote the nuclear factor erythroid 2–related factor 2 (Nrf2)/antioxidant responsive element (ARE) pathway.

## 1. Introduction

The liver is one of the most important organs in the body, performing up to 500 functions. It metabolizes most ingested substances and detoxifies toxic substances [1]. Hepatic injury, however, can be caused by the hepatotoxic effects of xenobiotics. Paracetamol (APAP; acetaminophen; *N*-acetyl-p-aminophenol) is a widely used drug worldwide due to its antipyretic property and is accessible in various formulations [2]. At therapeutic doses, APAP is considered a safe drug. It is also well-recognized, however, that APAP is responsible for acute liver failure [3]. In the United States, for example, about 30,000 patients annually are treated as a result of APAP hepatotoxicity [4]. 

In normal therapeutic doses, APAP is biotransformed in the liver to form glucuronide and water-soluble sulphate metabolites, which are excreted in the urine. In an overdose, however, APAP is oxidized by cytochrome p450 (CYP450) to form a highly reactive metabolite, *N*-acetyl-*p*-benzoquinone imine (NAPQI) that binds mainly to the sulfhydryl (-SH) group of GSH to form a complex of APAP-GSH, which excreted by the kidney as cysteine and mercapturic acid conjugates (APAP-cys). Furthermore, at toxic doses, APAP quickly depletes GSH, resulting in the accumulation of NAPQI, which then forms NAPQI-protein adducts as a result of covalent bond formation between NAPQI and –SH group of hepatocyte proteins [5]. The accumulation of these NAPQI-protein adducts results in turn in the formation of reactive oxygen species (ROS) that attack the cellular micro molecules causing lipid peroxidation and hepatic necrosis [6]. This damage to the hepatocytes ultimately leads to their leakage and a rise in hepatocyte enzymes in circulation in the blood. 

Several experimental studies reported on the protective role of medicinal plants against the hepatotoxicity induced by APAP [7,8], but there is still a need to evaluate other medicinal plants to see if they might offer effective alternatives. Nutmeg is a dry kernel of *Myristica fragrans* belonging to the family Myristicaceae, which is widely distributed in tropical countries. The seed is used as a spice or a flavour to food [9]. The non-volatile part of *M. fragrans* is rich in dimeric phenyl propanoids, lignans, and neolignans [10]. The anti-inflammatory [11], antihyperglycemic [12], and hepatoprotective role of *M. fragrans* [9] against isoproterenol (ISO)-hepatotoxicity have been studied previously. Furthermore, active constituents of nutmeg such as neolignan and myristicin have been reported to inhibit cytochrome CYP3A4 and CYP2C9 [13].

In this study, therefore, we explored the protective role of *M. fragrans* kernel extract against hepatotoxicity induced by APAP. Furthermore, the effect of *M. fragrans* kernel extract was compared with that of silymarin, a standard hepatoprotective agent.

## 2. Results

**Figure 1:**
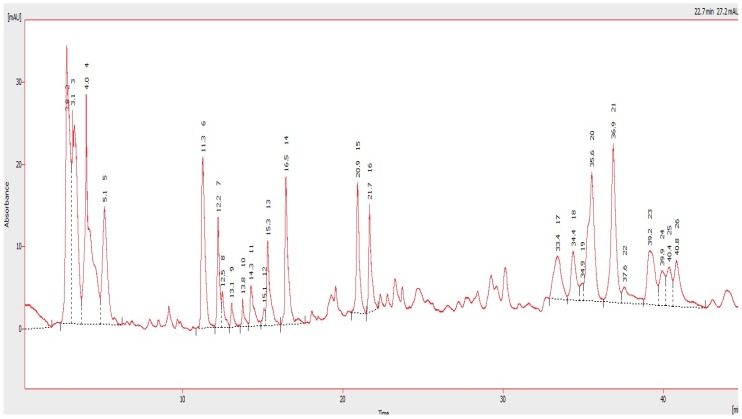
HPLC chromatogram of *Myristica fragrans* kernel extract at 280 nm. A mobile phase consisting of mixture of solvent A (0.2% acetic acid) and B (acetonitrile) and employing a gradient elution (from 10:90 to 100:0, *v*/*v*) at a flow rate of 1 mL/min.

The polyphenol and flavonoid fingerprint of the *Myristica fragrans* kernel extract detected at 280 nm is illustrated in Figure 1. The HPLC profile of MFKE shows the presence of 25 peaks with retention times ranging from 2.768 min to 40.842 min. Based on the UV-Visible spectral data and their retention times, the *Myristica fragrans* kernel extract has a UV band at 280 nm characteristic for polyphenol and flavonoid compounds, possibly caftaric acid and its derivatives, ellagic acid, rutin and catechin, and gallic acid and its derivatives, quercetin, and kaempferol.

**Figure 2:**
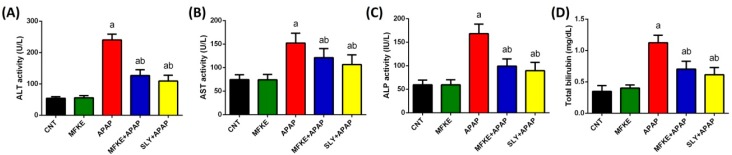
The effect of *Myristica fragrans* kernel extract (MFKE) on serum liver function markers in rats treated with paracetamol (APAP)-induced liver toxicity. Data are expressed as mean ± SD (*n* = 7); ^a^ *p* < 0.05 vs. control rats; ^b^ *p* < 0.05 vs. APAP-treated rats using Tukey’s post hoc test. (**A**) Alanine aminotransferase, (**B**) Aspartate aminotransferase, (**C**) Alkaline phosphatase and (**D**) Total bilirubin.

During the study, there was no prevalence of mortality and APAP administration did not cause any abnormal clinical signs, with no change in food and water consumption in the groups that received APAP. That said, the activity levels of serum liver function markers, namely, ALT, AST, ALP, and Total Bil, were significantly elevated in APAP-treated rats compared to the control rats (Figure 2). On the other hand, in the rats that had been pre-administered MFKE, the elevation in liver function markers was significantly less pronounced, indicating the hepatoprotective effect of *Myristica fragrans* kernels. Indeed, the hepatoprotective effect of MFKE was comparable to silymarin (SLY), which is widely used on account of its hepatoprotective properties.

**Figure 3:**
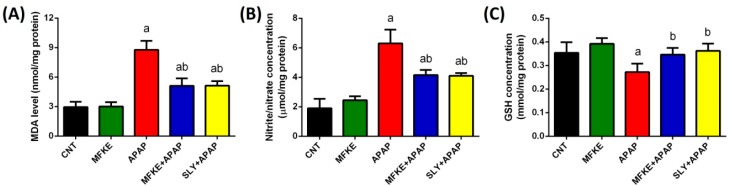
Effects of *Myristica fragrans* kernel extract (MFKE) on oxidative stress markers in rats treated with paracetamol (APAP)-induced liver toxicity. Data are expressed as mean ± SD (*n* = 7); ^a^ *p* < 0.05 vs. control rats; ^b^ *p* < 0.05 vs. APAP-treated rats using Tukey’s post hoc test. (**A**) lipid peroxidation, (**B**) nitric oxide, and (**C**) glutathione.

**Figure 4:**
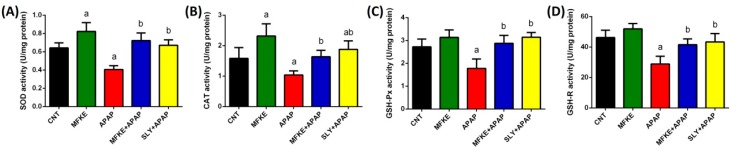
Effects of MFKE on the activity of antioxidant enzymes in rats treated with APAP-induced liver toxicity. Data are expressed as mean ± SD (*n* = 7); ^a^ *p* < 0.05 vs. control rats; ^b^ *p* < 0.05 vs. APAP-treated rats using Tukey’s post hoc test. (**A**) Superoxide dismutase, (**B**) Catalase, (**C**) Glutathione peroxidase, and (**D**) Glutathione reductase.

In order to evaluate the antioxidant effect of MFKE, lipid peroxidation, nitric oxide, and enzymatic and non-enzymatic molecules (GSH, SOD, CAT, GSH-Px, and GSH-R) were examined. SLY was used as a comparator. APAP treatment disturbed the redox status of hepatocytes confirmed by the elevation of LPO and NO (Figure 3), the depletion of GSH and the inhibition of the activities of antioxidant enzymes (Figure 4). On the contrary, MFKE pre-treatment significantly reversed this disturbance in the redox status. As expected, SLY pre-treatment also protected hepatocytes from oxidative stress induction by reversing the disturbance in the redox status. 

**Figure 5:**
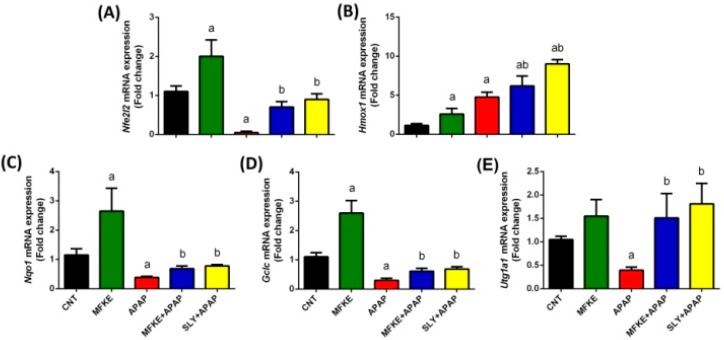
Effects of MFKE on Nrf2/ARE antioxidant signalling pathway gene expression in rats treated with APAP-induced liver toxicity. Results are presented as means ± SD of triplicate assays and normalized to *Gapdh* and expressed as fold change (log2 scale), relative to mRNA levels in controls; ^a^ *p* < 0.05 vs. control rats; ^b^ *p* < 0.05 vs. APAP-treated rats using Tukey’s post hoc test. (**A**) Nuclear factor erythroid 2–related factor 2, (**B**) Heme oxygenase 1, (**C**) NAD(P)H quinone oxidoreductase 1, (**D**) glutamate-cysteine ligase, catalytic, and (**E**) UDP glucuronosyltransferase family 1 member A1.

The study also examined *Nfe2l2* and the expression of its downstream target genes *Hmox1*, *Nqo1, Gclc*, and *Utg1a1*. APAP treatment in rats induced a significant decrease in *Nfe2l2* and its putative target genes compared to the control group, but *Hmox1* expression was significantly upregulated. In contrast, MFKE pre-treatment negated the APAP-induced impairment in the cellular detoxification system by enhancing *Nfe2l2*, *Hmox1*, *Nqo1*, *Gclc*, and *Utg1a1* mRNA expressions, and the treatment reduced the severity of the reduction compared to the APAP group (Figure 5). As expected, SLY pre-treatment was effective in protecting hepatic tissue from APAP-mediate toxicity.

**Figure 6:**
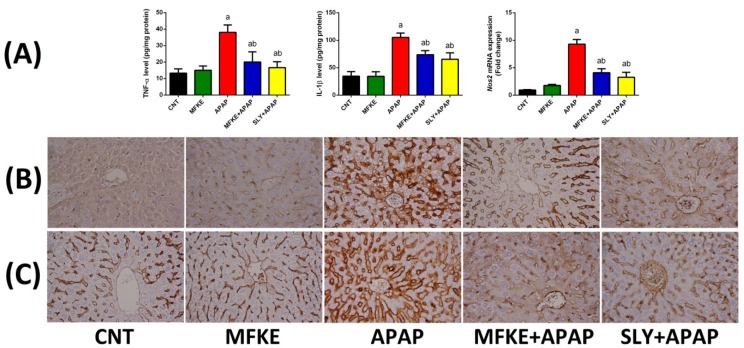
Effects of MFKE on proinflammatory biomarkers in rats treated with APAP-induced liver toxicity. (**A**) MRNA expressions of tumour necrosis factor-α, interlukin-1β and inducible nitric oxide synthase, (**B**) Nuclear factor ĸB protein expression in liver sections of different treated groups, and (**C**) Inducible nitric oxide synthase protein expression in liver sections of different treated groups. Original magnification ×40 for iNOS and NF-ĸB expression. Results for gene expression are presented as means ± SD of triplicate assays and normalized to *Gapdh* and expressed as fold change (log2 scale), relative to mRNA levels in controls; ^a^ *p* < 0.05 vs. control rats; ^b^ *p* < 0.05 vs. APAP-treated rats using Tukey’s post hoc test.

In the APAP group, inflammation was initiated and propagated through increases in the protein levels of TNF-α and IL-1β and in NF-κB and iNOS expressions (Figure 6). Pre-treatment with MFKE or SLY, however, prevented the overproduction of proinflammatory cytokines (TNF-α and IL-1β), and the protein expression of NF-κB and *Nos2* mRNA, suggesting that MFKE, like SLY, has an anti-inflammatory effect.

**Figure 7:**
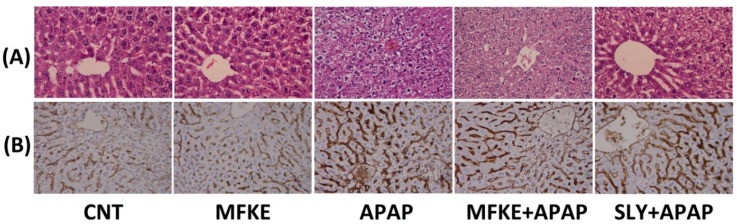
Effects of MFKE on liver pathology and α-SMA in rats treated with APAP-induced liver toxicity. (**A**) H&E stained photomicrographs of liver, and (**B**) Photomicrographs of α-SMA immunoreactivity in liver. Original magnification ×40.

The histopathological examination of the liver sections of the control and the rats treated with alone revealed a normal architecture of hepatic lobules with hepatic parenchyma radiating from the central veins, with narrow sinusoids and prominent nuclei. By contrast, microscopic examination of the APAP rats showed disruption of the normal architecture of hepatic lobules associated with granular degeneration of hepatocytes and infiltration of inflammatory cells accompanied with various degrees of centrilobular hepatocyte necrosis and central veins that showed severe congestion and dilation (Figure 7A). The rats pre-treated with MFKE (300 mg/kg), however, had much healthier hepatic tissue than the rats that had received only APAP, with scanty apoptotic hepatocytes and slight hepatocellular vacuolation. Rats pre-treated with SLY (50 mg/kg) also showed less severe hepatic injury than APAP with slight activation of Kupffer cells. Furthermore, APAP intoxication resulted in abundant activation of HSCs (hepatic stellate cells), as evidenced by an increase in alpha-smooth muscle actin (α-SMA) immunoreactive positive cells (Figure 7B). Pre-treatment with MFKE or SLY, however, successfully restrained the activation of HSCs by preventing α-SMA expression (Supplementary data: Table S2).

**Figure 8:**
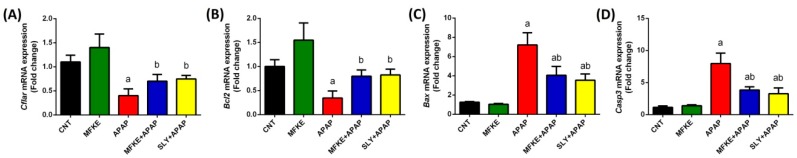
Effects of MFKE on apoptosis related gene expressions in rats treated with APAP-induced liver toxicity. Results are presented as means ± SD of triplicate assays and normalized to *Gapdh* and expressed as fold change (log2 scale), relative to mRNA levels in controls; ^a^ *p* < 0.05 vs. control rats; ^b^ *p* < 0.05 vs. APAP-treated rats using Tukey’s post hoc test. (**A**) Cellular FLICE (FADD-like IL-1β-converting enzyme)-inhibitory protein, (**B**) B-cell lymphoma 2, (**C**) BCL2 Associated X, and (**D**) Caspases 3.

In order to uncover the mechanism through which MFKE mitigates APAP-induced liver injury, we used qRT-PCR to examine the expression levels of anti-apoptotic markers, including *Bcl2* and *Cflar*, and pro-apoptotic markers *Bax* and *Casp3* in liver tissue. Our results showed a significant downregulation in *Bcl2* and *Cflar* expressions after APAP injection, but a significant upregulation in *Bax* and *Casp3* expressions (Figure 8). In contrast, the MFKE pre-treatment obviously reduced the number of hepatocytes dying as a result of APAP-injection. The hepatoprotective effect of MFKE was comparable to SLY. Overall, our results indicate that MFKE pre-treatment ameliorates damage to hepatocytes by inducing anti-apoptotic genes and restraining pro-apoptotic genes.

## 3. Discussion

APAP is an over-the-counter (OTC) drug commonly prescribed for its antipyretic and analgesic effects. In overdose, however, APAP can cause acute liver failure [6]. In the present study, injection with APAP caused liver injury, evidenced by an increase in liver function markers and pathological changes to liver tissue. In fact, ALT, AST, ALP, and total Bil are routinely monitored to assess liver function and any increase of these enzymes in serum is considered as indicative of liver injury and dysfunction. The increase in these markers indicates necrosis of hepatocytes or an increase in the permeability of their membranes. However, Stec et al. [14] reported that transaminases reduced significantly with the antioxidant properties of bilirubin. In addition, Hinds et al. [15] considered bilirubin as an antioxidant as well as an anti-inflammatory biomolecule and mice expressing the human Gilbert’s HuUGT*28 polymorphism are safe from high-fat diet-induced hepatic steatosis and insulin resistance due to an elevation of bilirubin. Our biochemical data were also confirmed by histopathological observations which showed obvious hepatocyte degeneration in APAP-treated rats, as well as severe haemorrhage and dilation in sinusoids accompanied by Kupffer and inflammatory cells. MFKE pre-treatment, however, successfully prevented this liver damage. The hepatoprotective effect of MFKE indicates that MFKE is capable of maintaining the membrane integrity of hepatocytes and preventing the leakage of liver enzymes, thereby safeguarding liver function even when challenged by APAP overdose. The hepatoprotective efficacy of MFKE was previously shown in lipopolysaccharide/D-galactosamine-induced liver injury and inflammation [16] and in radiation-induced liver toxicity and oxidative stress [17]. Interestingly, the hepatoprotection offered by MFKE is comparable to the standard hepatoprotector SLY, with both of them showing clear preventative effects histologically.

Furthermore, oxidative stress induced by APAP injection was evidenced by significant elevation in LPO and NO, concurrently with a significant depletion in GSH and inhibition of the antioxidant defence system. APAP is oxidized mainly by the P450 enzyme, and it is this enzyme that is responsible for oxidative stress induction in the liver [5]. Moreover, the previous study reported that many mitochondrial proteins are targeted by APAP metabolites, including GSH-Px and ATP-synthase alpha-subunit, which are adducted by NAPQI. As a result, 60% of GSH-Px was modified and ATP-synthase function was impaired causing cessation in ATP production. Additionally, NAPQI interferes with the mitochondrial electron transport chain causing the release of electrons from the chain which triggers ROS generation and lipid peroxidation [18]. Furthermore, the escaped electrons from the chain react with oxygen to form superoxide anion, which could either dismutate to form H_2_O_2_ or react with NO to form peroxynitrite, a strong oxidant. The formed H_2_O_2_ can be detoxified by GSH, while peroxynitrite is harsher and reacts with a variety of biomolecules and alerted biomolecules’ structure [19]. Thus, the overproduction of H_2_O_2_ and peroxynitrite, in addition to the other ROS, can overwhelm the antioxidant defence system.

The overproduction of NO in the present study is attributed to the upregulation of iNOS in liver tissue. iNOS is another source of ROS in inflammatory cells and iNOS blockers have been found to inhibit APAP-induced hepatotoxicity [20]. Interestingly, promoting the replenishment of endogenous antioxidant molecules with natural products is effective at protecting the liver from APAP overdose-induced hepatotoxicity [21].

In this study, the induction of hepatotoxicity by APAP caused significant increases in TNF-α and IL-1β level and iNOS expression. There is growing evidence to show that inflammation is also a possible mechanism responsible for the pathogenesis resulting from APAP-induced hepatotoxicity. Furthermore, both inflammation and oxidative stress are tightly linked to each other in that, at the site of inflammation, inflamed cells like macrophages are releasing ROS, causing exaggerated oxidative damage. Furthermore, overproduction of ROS initiates a cellular signalling cascade that promotes the gene expression of proinflammatory cytokines [22]. Moreover, Posadas et al. [23] demonstrated that APAP promoted NF-κB p65 phosphorylation at Ser536 and enhanced NF-κB p65 translocation to the nucleus, a contention that supports our findings. Indeed, NF-κB p65 nuclear translocation has been found to induce a variety of proinflammatory cytokines and mediators. The results obtained in our study in respect to the effect of APAP on the liver therefore are generally in line with those of previous studies, and support the existing understanding of the mechanism of action of APAP on liver tissue and cells [20,24].

Meanwhile, the current study demonstrated that pre-treatment with MKFE restrained the oxidative stress and inflammation otherwise induced by APAP, as evidenced by significant decreases in LPO, NO and proinflammatory cytokine levels, as well in NF-κB and iNOS expressions, and significant increases in enzymatic and nonenzymatic molecules. This effect suggests that MFKE has antioxidant properties that might be attributed to the presence of different phenolic compounds, flavonoids, alkaloids, and anthraquinones [25]. It was reported that argenteane, a dilignan which was isolated from MFKE, is as powerful as vitamin E. Indeed, argenteane shows a typical free radical scavenging activity by releasing one or two H atom(s) to the medium [26]. Furthermore, Kapoor et al. [27] demonstrated that essential oil and oleoresins from nutmeg are antioxidants similar to the synthetic butylated hydroxyanisole (BHA) and butylated hydroxytoluene (BHT). Most recently, Erukainure et al. [28] found that more than 14% of nutmeg is oleic acid, which is known for its antioxidant activity, and more than of 46% of the extract is 17-octadecynoic acid, which inhibits cytochrome P450. Additionally, MFKE contains myristicin, which was found to possess antioxidant and hepatoprotective activities [16] and to suppress lipid peroxidation in the liver by trapping free radicals or ROS directly [29]. Furthermore, at high doses (96 mg/kg), pure myristicin is a potent enhancer of hepatic cytochrome P enzymes, causing a 2–20 fold increase in the activity and expression of these enzymes [30]. 

The myristicin in MFKE does not only act as an antioxidant agent, but also as an anti-inflammatory agent. The anti-inflammatory effect of this constituent was observed in an in vitro study using RAW 264.7 mouse macrophages. In this study, myristicin suppressed the production of different proinflammatory cytokines [31]. Further consideration indicated that this molecule played a regulatory role in chronic autoimmune diseases and that it also attenuated lung inflammatory disease by inhibiting NO production [32]. Moreover, MFKE is a rich source of neolignans that have showed potent NF-κB inhibitory activity [33].

Paracetamol-induced liver fibrosis received growing attention since the reports of occurrences of liver fibrosis and cirrhosis in patients using APAP to reduce pain [34], and the activation of HSCs plays an important role in liver fibrosis. Indeed, HSCs are considered to be the main extracellular matrix–producing cells in the liver [35]. α-SMA expression is a typical marker for activated HSCs [36], and Shen et al. [37] demonstrated that α-SMA expression caused HSCs activation induced acute liver failure. Our study is in line with this work in that we reported that APAP intoxication caused α-SMA expression in the liver.

It is therefore significant that are our results also show that MFKE successfully restrained the activation of HSCs by preventing α-SMA expression. The possible mechanism for this effect might be linked to the presence of meso-dihydroguaiaretic acid, which directly inhibits transactivation of HSCs through downregulating transforming growth factor beta1 and inhibiting activator protein 1 activity [38].

In addition, in the current study, APAP treatment caused a significant upregulation in the HO-1 gene and downregulation in Nrf2 expression. Nrf2 is a prime controller of intracellular redox homeostasis by controlling the antioxidant response element (ARE), which orchestrates adaptability to cellular redox disruption. Chan et al. found that, in Nrf2-knocout mice, APAP intoxication enhanced liver injury and mortality compared with wild-type mice [39]. In this context, the ability of MFKE to promote Nrf2 expression in the present study implied the cytoprotective effect of nutmeg. One way in which it may achieve this is through nectandrin B. Song et al. [40] and Kim et al. [41] demonstrated that nectandrin B, which was isolated from nutmeg, protected hepatocytes against oxidative perturbation through the activation of Nrf2 and the consequent induction of the detoxifying antioxidant enzymes such as NQO1, GCLC, and UTGs. NQO1 is a highly inducible enzyme responsible, among others, for a single-step two-electron reduction of quinones and quinone imines, thus inhibiting the formation of reactive and toxic semiquinone intermediates [42]. Meanwhile, GCLC is the first rate-limiting enzyme complex for GSH synthesis [43]. UGTs play predominant roles in the detoxification of many exogenous and endogenous agents by forming more polar and water-soluble glucuronides [44] and deficiencies in expression of UGTs are responsible for APAP hepatotoxicity observed in rats [45]. HO-1, meanwhile, is the primary rate-limiting enzyme in heme catabolism and is well known to have a cytoprotection effect against liver damage by restraining oxidative stress and inflammation. HO-1 is also involved in maintaining the oxidants/antioxidants balance [46] by increased formation of the antioxidant, bilirubin [15]. In some conditions, however, induction of HO-1 may cause damage and enhance cytotoxicity by increasing the products of heme degradation, such as iron ions, biliverdin, and carbon monoxide [47]. Furthermore, biliverdin is subsequently converted to bilirubin this has been recognized to be an antioxidant and can inhibit lipid peroxidation. The higher plasma bilirubin levels within the normal range were linked with a significant and marked reduction in cardiovascular diseases risk and decreased the incidence of ischemic heart disease [48]. In the present study, APAP treatment induced upregulation in HO-1 expression. Our results are in harmony with those of Bauer et al. [49] and Gao et al. [50]. MFKE pre-treatment, however, modulated HO-1 expression and induced significant down-regulation in the gene compared to the APAP group. When comparing the HO-1 expression with the control group, however, HO-1 showed significant increase in the MFKE and SLY treatment groups. As we expected that silymarin and nutmeg extract as targeted Nrf2 would lead to UGT1A1 upregulation, and this enzyme is the sole enzyme responsible for the bilirubin metabolism, that may explain the decrease in bilirubin in APAP-treated groups. However, silymarin and many flavonoids are known to inhibit UTGs in vitro assays. Those in vitro results turned out to be ineffective in vivo studies due to the poor cell permeability and poor metabolic stability of these natural compounds, together leading to poor bioavailability, are probably the major causes of their ineffectiveness in vivo [44] that seen herein.

The results obtained in the present study revealed that APAP intoxication caused severe apoptosis and necrosis in liver tissue. The death of hepatocytes was evidenced by a significant elevation of pro-apoptotic genes (Bax and caspases-3) and a significant reduction in anti-apoptotic genes (c-FLIP and Bcl-2). It was reported that overdose with APAP induced apoptosis in primary hepatocytes [51] and liver tissue of mice [52]. Moreover, hepatocyte apoptosis leads to elevated serum transaminases levels and further reduction of the liver function and preventing apoptosis inhibits liver failure [53]. The precise mechanisms by which APAP-induced apoptosis are linked to the ability of APAP to induce mitochondrial permeability transitions that are subsequently associated with the release of cytochrome c, second mitochondria-derived activator of caspase (Smac) and apoptosis inducing factor (AIF) [54]. Once in cytosol, cytochrome c binds to the adaptor protein Apaf-1 thus triggering the formation of apoptosome by activating caspases-9. Meanwhile, AIF translocates to the nucleus causing DNA damage due to its involvement in DNA fragmentation. Smac, meanwhile, plays a vital role in caspase-3 activation [55]. This induction of apoptosis in the liver was alleviated in the rats treated with MFKE, however, with the liver tissue of these rats showing marked improvement compared to the rats treated just with APAP. To understand this anti-apoptotic effect of MFKE, it can be noted that, in the current study, MFKE administration significantly upregulated c-FLIP. The cellular Fas-associated death domain-like IL-1β-converting enzyme-inhibitory protein is known as c-FLIP, and this is a master anti-apoptotic regulator with a multifunctional role in several signalling pathways, as well as in inducing several cytoprotective and pro-survival signalling pathways [56]. The anti-apoptotic effect of MFKE might, therefore, be related to the ability of myristicin and nectandrin B to inhibit endoplasmic reticulum stress or to promote c-FLIP expression and thus enhance the antioxidant defence system [40,57].

In conclusion, our findings highlight a body of evidence for the hepatoprotective effect of *Myristica fragrans* kernels in respect to the liver of rats exposed to APAP-induced hepatotoxicity. These favourable effects were mediated via suppressing oxidative stress, inflammation, and apoptosis, and this hepatoprotection effect could be linked to their ability to promote the Nrf2/ARE pathway. Interestingly, the hepatoprotective effect of MFKE was comparable to that exerted by the reference hepatoprotector SLY.

## 4. Materials and Methods

### 4.1. Chemicals

APAP and silymarin were obtained from Sigma-Aldrich (St. Louis, MO, USA). All other chemicals and reagents used in the experiment were of analytical grade.

### 4.2. Plant Materials and Extraction Procedure

The kernels of *Myristica fragrans* (MF) were obtained from a local market in West-Cairo, Egypt. The obtained kernels were identified by an expert taxonomist from the Botany Department, Faculty of Science, Helwan University, Egypt. The kernels were ground into powder using an electrical blender and extracted three times by maceration with 70% methanol. The ratio of nutmeg kernels to the solvent was 1:10 (*w*/*v*). The solvent was concentrated under a vacuum evaporator and subsequently lyophilized. The *Myristica fragrans* kernel extract (MFKE) was kept at −20 °C until used in the current experiment. Total phenolic and flavonoid compounds were determined as described previously [58]. The amount of the total phenolic compounds was 21.3 mg gallic acid equivalent/g extract, while the amount of flavonoid compounds was 20.8 mg quercetin equivalent/g extract.

### 4.3. HPLC Analysis

High performance liquid chromatography (HPLC) analysis was performed using a Perkin Elmer Series 200 liquid chromatography (PerkinElmer, Akron, OH, USA) to determine the polyphenol and flavonoid constituents of MFKE. The HPLC column was an AQUA column 150 mm 5 μ C18 (Phenomenex), with a detection wavelength of 280 nm. Elution was carried out using acetic acid (2%; A) and acetonitrile (B). The flow rate was set at 1 mL/min throughout the elution.

### 4.4. Animals

Adult male Wistar albino rats (10 weeks old, weighing 200–220 g) obtained from VACSERA (Cairo, Egypt) were used in the current experiments. The animal housing conditions was as described previously [59]. Briefly, the rats were housed in wire cages made from polypropylene in a room under standard laboratory conditions (12 h light-dark cycle; 25 ± 2 °C). A standard rodent diet (El Gomhorya Company, Ismailia, Egypt) and water were available *ad libitum*. The rats were acclimated to the environment for one week before the beginning of the experiment. The study was approved by the Committee of Research Ethics for Laboratory Animal Care, Department of Zoology, Faculty of Science, Helwan University (24/09/2018, Cairo; approval no, HU2017/Z/08) and was conducted according to the European Community Directive (86/609/EEC), the national rules on animal care and in accordance with the NIH Guidelines for the Care and Use of Laboratory Animals, eighth edition.

### 4.5. Experimental Design

The animals were fasted for 24 h prior to the experiment under standard laboratory conditions but allowed access to distilled water (dH_2_O) *ad libitum*. Subsequently, the animals were randomly divided into five experimental groups of seven rats each and administered with test solutions orally once daily for seven consecutive days, as follows. Group I, serving as the normal control, received 10 mL/kg physiological saline (0.9% NaCl). Group II, serving as the MFKE control, received 300 mg/kg of MFKE. Group III, serving as the APAP (paracetamol) control group received 2 g/kg. Groups IV and V, pre-treatment groups, received 300 mg/kg MFKE and 50 mg/kg SLY (silymarin), respectively. All the treatments were suspended or dissolved in a physiological saline.

An acute toxicity study was performed using a maximum dose of 2000 mg/kg MFKE administered orally and observed in order to assess changes in skin and fur, eyes, and mucous membranes, and also respiratory, circulatory, autonomic and central nervous systems, and somatomotor activity and behavior pattern. This dose, after 14 days, did not lead to any signs of toxicity in the rats, and the subsequent oral dose of MFKE was selected based on a preliminary study using three doses of 100, 200, and 300 mg/kg, which showed that the oral administration of MFKE at a dose of 300 mg/kg effectively prevented APAP-induced hepatotoxicity. While, APAP dose was based on the previous studies to induce liver toxicity in rats by using APAP at 2 g/kg [60,61].

The oral administration of APAP (2 g/kg) was performed three hours prior to the last administration on the seventh day except for groups I and II, which received only 10 mL/kg physiological saline. Forty-eight hours after the induction of hepatic injury by these means, the animals were lightly anesthetized using an appropriated anaesthetic agent and blood was collected by cardiac puncture in sterilized centrifuged tubes which were then centrifuged at 3000 rpm for 10 min to get serum for the study of biochemical parameters. The animals were then killed by cervical dislocation and the liver was quickly removed, weighed and washed clean of blood with ice-cold saline. Subsequently, a small piece was homogenized in cold phosphate buffer (0.05 M, pH 7.4), and then centrifuged at 3000 rpm for 10 min at 4 °C. The resulting supernatant was used for determination of biochemical markers. The remaining parts of the liver were used for the histopathological and molecular studies.

### 4.6. Biochemical Parameters

#### 4.6.1. Liver Functions Tests

A colorimetric method was used to determine the activity of alanine transaminase (ALT) and aspartate transaminase (AST) enzymes in the collected serum [62] using kits obtained from Bio-Diagnostic (Giza, Egypt) the results were recorded and analysed using an UV-visible spectrophotometer (V630; JASCO, Tokyo, Japan) at 505 nm. Alkaline phosphatase and total bilirubin (TB) in serum were estimated according to the methods described by Shephard and Peake [63] and Schmidt and Eisenburg [64] using kits obtained from Bio-Diagnostic (Giza, Egypt) and Randox (Crumlin, UK). The results were recorded and analyzed using an UV-visible spectrophotometer (V630; JASCO, Japan) at 510 and 535 nm, respectively.

#### 4.6.2. Determination of Malondialdehyde and Nitric Oxide

Hepatic malondialdehyde (MDA) was estimated using 1 mL of 0.67% thiobarbituric acid according to the method of Ohkawa et al. [65]. Meanwhile, the level of nitrite/nitrate [nitric oxide (NO)] was determined according to the method described by Green et al. [66].

#### 4.6.3. Nonenzymatic and Enzymatic Antioxidant Molecules

Hepatic glutathione (GSH) was assayed spectrophotometrically using the Ellman [67] method. Determination of superoxide dismutase (SOD) and catalase (CAT) activities were assayed according to the methods described by Sun et al. [68] and Luck [69], respectively. In addition, the activity of glutathione related enzymes, namely, glutathione peroxidase (GSH-Px) and glutathione reductase (GSH-R), were assayed according to the techniques described by Paglia and Valentine [70] and Factor et al. [71], respectively.

#### 4.6.4. Determination of Proinflammation Markers

The hepatic proinflammatory cytokines, namely, interleukin-1 beta (IL-1β; Cat. no. ERIL1B, ThermoFisher Scientific, Waltham, MA, USA) and tumour necrosis factor alpha (TNF-α; Cat. no. CSB-E11987r, CUSABIO Life Sciences, Wuhan, China), were assayed using enzyme-linked immunosorbent assay (ELISA) kits according to the manufacturer’s instructions. Each liver homogenate sample was measured in duplicate.

#### 4.6.5. Quantitative Reverse Transcription-Polymerase Chain Reaction (qRT-PCR) Analysis

Total RNA was isolated from liver samples using TRIzol reagent (Invitrogen, Carlsbad, CA, USA) and reverse transcribed to complementary DNA (cDNA) using the Script™ cDNA synthesis kit (Bio-Rad, California, CA, USA). Quantitative real-time polymerase chain reaction (qRT-PCR) was performed using Power SYBR^®^ Green (Invitrogen) on an Applied Biosystems 7500 Instrument. The cycling conditions for amplification were 95 °C for 2 min, followed by 40 cycles at 94 °C for 60 s and 60 °C for 90 s. Each assay was performed in duplicate to determine delta CT. Specific primers were purchased from Jena Bioscience GmbH (Jena, Germany). The housekeeping gene was glyceraldehyde-3-phosphate dehydrogenase (GAPDH). The primer sequences for *Gapdh*, *Nos2*, *Nfe2l2*, *Nqo1*, *Gclc*, *Utg1a1*, *Hmox1*, *Cflar*, *Bcl2*, *Bax*, and *Casp3* were listed previously [72] (see Supplementary data: Table S1).

### 4.7. Histopathological Examination

The liver tissues were fixed in 10% neutral-buffered formalin for 24 h at room temperature, dehydrated in gradual ethanol, embedded in paraffin wax, and sectioned (4–5 μm). The deparaffinized sections were stained consistently with haematoxylin and eosin dye for microscopic examination. Images were recorded with 400× magnification (Nikon Eclipse E200-LED, Tokyo, Japan).

### 4.8. Immunohistochemistry Analysis

To investigate NF-κB, iNOS and αSMA, the paraffinized sections were blocked with hydrogen peroxide (0.1%) containing methanol for 15 min to damage the endogenous peroxidase. After blocking, the samples were incubated with the primary antibodies at 4 °C overnight. Thereafter, the samples were washed twice with phosphate-buffered saline and incubated with biotinylated secondary antibody labelled with horseradish peroxidase (HRP). The reactions were developed via an HRP-catalysed reaction with diaminobenzidine (DAB), followed by counterstaining with haematoxylin. Images were recorded at 400× magnification (Nikon Eclipse E200-LED, Tokyo, Japan). Afterward, the color intensity of each examined protein was semi-quantitatively scored by a blinded pathologist. The intensity was expressed as + (weak immunoreaction), ++ (moderate immunoreaction), +++ (strong immunoreaction), or ++++ (very strong immunoreaction).

### 4.9. Statistical Analysis

Data obtained are expressed as the mean ± standard deviation. Data from various evaluations were analysed using one-way analysis of variance (ANOVA) and the differences between the groups were determined using Tukey’s post hoc test; *p* values < 0.05 were taken to be statistically significant.

## Supplementary Materials

Supplementary materials can be found at https://www.mdpi.com/1422-0067/20/4/993/s1.
