# Supplementary material for: Myristica fragrans Kernels Prevent Paracetamol-Induced Hepatotoxicity by Inducing Anti-Apoptotic Genes and Nrf2/HO-1 Pathway
Source: Int J Mol Sci. 2019 Feb 25;20(4):993. doi: 10.3390/ijms20040993 (PMC6412641; doi:10.3390/ijms20040993)
Supplement: Supplementary file 1 [file ijms-20-00993-s001.pdf]

Table S1. Primer sequences of genes analyzed by real-time PCR

Name	Accession number	Sense (5'---3')	Antisense (5'---3')
Gapdh	NM_017008.4	AGTGCCAGCCTCGTCTCATA	GATGGTGATGGGTTTCCCGT
Nos2	NM_012611.3	GTTCCTCAGGCTTGGGTCTT	TGGGGGAACACAGTAATGGC
Nfe2l2	NM_031789.2	TTGTAGATGACCATGAGTCGC	ACTTCCAGGGGCACTGTCTA
Nqo1	NM_017000	ATTGTATTGGCCCACGCAGA	TCATATCCCAGGCCACCTGA
Gclc	NM_012815.2	TGTCGCTGGGGAGTGATTTC	GGTCAGACTCGTTGGCATCA
Utg1a1	NM_012683.2	CTGTGTGTGTTGGGTCCCTC	GCCTCAGCAATTTCCATCGC
Hmox1	NM_012580	TTAAGCTGGTGATGGCCTCC	GTGGGGCATAGACTGGGTTC
Cflar	NM_001033864.3	GAGCCTCTCAAGAAGCCACT	CTCAGCAGACACAGTGCTCA
Bcl2	NM_016993	ACTCTTCAGGGATGGGGTGA	TGACATCTCCCTGTTGACGC
Bax	NM_017059.2	GGGCCTTTTTGCTACAGGGT	TTCTTGGTGGATGCGTCCTG
Casp3	NM_012922.2	GAGCTTGGAACGCGAAGAAA	TAACCGGGTGCGGTAGAGTA

Abbreviations; GAPDH: Glyceraldehyde-3-phosphate dehydrogenase; Nos2: Inducible nitric oxide synthase; Nrf2: Nuclear factor erythroid 2–related factor 2; Nqo1: NAD(P)H quinone oxidoreductase 1; Gclc: Glutamate-cysteine ligase, catalytic; Utg1a1: UDP glucuronosyltransferase family 1 member A1; Hmox1: heme oxygenase 1; Cflar: cellular FLICE (FADD-like IL-1β-converting enzyme)-inhibitory protein ; Bcl-2: B-cell lymphoma 2; Bax: Bcl-2-associated X protein.

Table S2: Effects of *Myristica fragrans* kernel extract (MFKE) on the immunohistochemistry intensity of nuclear factor κB (NF-κB), inducible nitric oxide synthase (iNOS) and alpha-smooth muscle actin (α-SMA) in rats treated with paracetamol (APAP)-induced liver toxicity.

Apoptosis protein	NF-κB	iNOS	α-SMA
**CNT**	+	+	+
**MFKE**	+	+	+
**APAP**	+++	+++	+++
**MFKE+ APAP**	++	++	++
**SLY+ APAP**	+	++	++

Note: + = weak immunoreaction, ++ = moderate immunoreaction, +++ = strong immunoreaction, or ++++ = very strong immunoreaction.
